# Workplace stress: prevalence, sources, and consequences among academic staff at the University of Calabar, Nigeria

**DOI:** 10.3389/fpubh.2026.1872922

**Published:** 2026-06-23

**Authors:** Regina Idu Ejemot-Nwadiaro, Nuria Sylvanus Nwachuku, Eucharia Obiageli Obiekezie, Joshua Pam Mwankon, Elizabeth Libuo-Beshel Nji, Peter Bassey Enyievi, Okechukwu Paul-Chima Ugwu

**Affiliations:** 1 Directorate of Research, Innovation, Consultancy and Extension, Kampala International University, Kampala, Uganda; 2 Department of Public Health, University of Calabar, Calabar, Nigeria; 3 Arts Education Department, College of Education, University of Calabar, Nigeria; 4 Department of Family Medicine, University of Calabar Teaching Hospital, Calabar, Nigeria; 5 Department of Public Health and Community Medicine, Kampala International University in Tanzania, Dar es Salaam, Tanzania; 6 Department of Publication and Extension, Kampala International University, Kampala, Uganda

**Keywords:** academic staff, asset, hypertension, job satisfaction, Nigeria, occupational stressors, work productivity, workplace stress

## Abstract

**Background:**

Workplace stress among university academics in sub-Saharan Africa remains insufficiently investigated, particularly in studies that combine validated psychometric assessment with objective clinical measurements and evaluation of stress-related lifestyle behaviours. This study addressed this gap by examining workplace stress and associated factors among academic staff at the University of Calabar (UNICAL), Nigeria.

**Methods:**

A cross-sectional survey was conducted among 401 permanently employed academic staff drawn from all 15 faculties of UNICAL. Sample size was calculated using the Kish formula (Z = 1.96, *p* = 0.75, d = 0.05), incorporating finite population correction, design effect, and a 15% non-response allowance, yielding a minimum target of 364 participants. Systematic random sampling was used within faculties. Workplace stress was assessed using the validated A Shortened Stress Evaluation Tool (ASSET), with scores classified as low (≤142), moderate (143–231), or high (≥232). Objective anthropometric and physiological measurements were collected alongside self-reported data. Associations were examined using chi-square tests, with statistical significance set at *p* < 0.05.

**Results:**

Workplace stress was highly prevalent, with 84.3% of respondents classified within the moderate ASSET stress category and 15.0% within the high category. Self-reported stress was significantly associated with gender, educational attainment, academic rank, and teaching experience (all *p* < 0.001). Using ASSET-derived stress categories, significant associations remained for educational level, rank, and experience (all *p* < 0.001), whereas gender was not significant (*p* = 0.145). Work overload emerged as the leading productivity-related stressor (15.2% first-ranked), while inadequate pay and benefits was the principal job satisfaction stressor (17.7% first-ranked). High-stress respondents reported more frequent alcohol consumption (χ^2^ = 9.84, *p* = 0.043), lower physical activity levels (χ^2^ = 11.27, *p* = 0.024), and greater hypertension medication use. No significant association was observed between BMI category and ASSET stress level (χ^2^ = 2.17, df = 4, *p* = 0.704).

**Conclusion:**

Workplace stress is pervasive among UNICAL academics and is linked to adverse lifestyle behaviours and hypertension. Institutional interventions should prioritise workload management, equitable remuneration, career advancement opportunities, and access to mental health and occupational wellness services.

## Introduction

Occupational stress has become one of the more pressing public health challenges of our time. It cuts across professions and geographies, and the academic sector is no exception. University lecturers in many parts of the world now work under conditions that would strike previous generations of academics as almost unrecognizable balancing mounting teaching loads, publication demands, student supervision, administrative responsibilities, and the chronic uncertainty of funding cycles. That said, the consequences are not uniformly distributed: the structural and financial constraints facing Nigerian universities sit in a different register entirely from those confronting their counterparts in high-income countries.

Workplace stress, as defined by the World Health Organization (1), occurs when demands and pressures at work exceed an individual’s knowledge, abilities, and capacity to cope. The evidence linking such stress to diminished job satisfaction, reduced productivity, and increased occupational accidents is well established (2–4, 25). What is perhaps less appreciated is the magnitude of physiological consequences: prolonged occupational stress is now firmly linked to hypertension, diabetes, depression, and broader non-communicable disease burden (4, 5).

Higher education in sub-Saharan Africa presents a particularly acute set of structural stressors. Chronic underfunding, rapid enrolment growth without proportional resource investment, staff shortages, deteriorating physical infrastructure, and inconsistent remuneration create a working environment that is objectively demanding (6, 7). Nigerian universities are no different. Expanding student numbers without matching increases in staffing or facilities has intensified workloads, eroded work–life balance, and generated institutional uncertainty that undermines academic morale (7, 8). The consequences extend beyond individual wellbeing: institutional productivity, educational quality, and national human capital development are all downstream casualties (9).

Despite this, most published studies from the Nigerian context have relied on non-standardised instruments, presented descriptive lifestyle data in isolation from stress levels, and rarely integrated objective clinical measurements with psychosocial data (11). The ASSET (A Shortened Stress Evaluation Tool) (10, 11) addresses these limitations. Developed and validated against a normative database of over 9,000 workers, ASSET provides psychometrically robust assessment across 12 organisational stress domains and produces total scores benchmarked to empirically derived population categories. Its application in this context offers a standardised basis for comparison with international evidence.

The present study makes four specific contributions relative to prior Nigerian work. First, it employs ASSET with its established normative thresholds rather than non-validated instruments. Second, it integrates objective anthropometric and physiological measurements body mass index (BMI), blood pressure, waist and hip circumferences alongside psychosocial data. Third, it explicitly examines associations between ASSET stress category and lifestyle behaviours (alcohol consumption, physical activity), moving beyond the purely descriptive approach of earlier studies. Fourth, it links stress levels to self-reported health outcomes including medication use for hypertension. The study aimed to: (a) determine the prevalence and distribution of workplace stress among UNICAL academic staff; (b) identify principal organisational stressors affecting job productivity and satisfaction; (c) examine associations between stress and lifestyle behaviours; (d) investigate relationships between stress levels and objective and self-reported health outcomes; and (e) generate an evidence base for institutional occupational health policy.

## Methods

### Study area

The University of Calabar (UNICAL), established in 1975, is a public research institution in Calabar Municipality Local Government Area, Cross River State, Nigeria (4.50°N, 8.30°E). It comprises approximately 15 faculties and serves an estimated student population of 44,000, supported by approximately 3,500 permanent academic staff—a student-to-staff ratio of roughly 13:1.

### Study design

A quantitative cross-sectional design was used. The study was framed within the Social Ecological Model, which conceptualises health outcomes as products of individual, interpersonal, organisational, and community-level factors interacting within physical and social environments. This framework was selected because workplace stress in Nigerian universities is shaped simultaneously by personal characteristics, peer dynamics, organisational culture, and broader socio-economic conditions.

### Sample size and sampling

Sample size was calculated using the Kish (12) formula for estimating proportions: *n* = Z^2^P(1 − P)/d^2^, where Z = 1.96 (95% confidence), *p* = 0.75 [estimated baseline prevalence of moderate-to-high occupational stress among Nigerian university academics, derived from Akah et al. (7) and Orunbon et al. (8)], and d = 0.05 (precision margin). This produced *n* ≈ 288. After applying a finite population correction for *N* = 3,500 and a design effect of 1.2 to account for the clustered multi-faculty sampling structure, and adding a 15% non-response allowance, the minimum target was 364. The enrolled sample of 401 exceeded this comfortably. Faculty-level representation was ensured through equal allocation across all 15 faculties (27 participants per faculty), with participants selected by systematic random sampling from each faculty’s staff register.

### Instrument

A structured, self-administered questionnaire comprised four sections. Section A captured socio-demographic, anthropometric, and professional data. Sections B–D incorporated items adapted from the validated ASSET (10, 11), assessing perceived stressor burden across 12 organisational domains, psychological and physical well-being, and organisational commitment. ASSET total scores were categorised as low (≤142), moderate (143–231), or high (≥232) using empirically established normative thresholds (11). Internal consistency was satisfactory (Cronbach’s *α* = 0.803). The instrument was pre-tested on 20 academic staff at the Cross River University of Technology (CRUTECH) a comparable state-owned institution deliberately chosen to avoid contaminating the UNICAL study population and to ensure contextual transferability.

### Clinical measurements

Seated blood pressure was recorded using a mercury sphygmomanometer after at least 15 min’ rest, with two readings taken. Hypertension classification followed the Eighth Joint National Committee (JNC 8) guidelines (13). Body weight (kg) and height (cm) were measured to calculate BMI; waist and hip circumferences (cm) were recorded for body fat distribution characterisation.

### Data analysis

Data were entered and analysed in Epi Info^™^ version 7. Continuous variables are presented as means and standard deviations (SD); categorical variables as frequencies and proportions. Chi-square tests examined associations between ASSET category and socio-demographic characteristics, lifestyle behaviours (alcohol consumption frequency, physical activity level), and health outcomes (hypertension medication use). One-way ANOVA and Student’s t-test were applied to continuous outcomes where relevant. Statistical significance was set at *p* < 0.05 throughout.

### Ethics

Ethical approval was granted by the Health Research Ethics Committee of the University of Calabar Teaching Hospital (UCTH/HREC/33/867), and institutional permission was obtained from UNICAL management. The study adhered to the Declaration of Helsinki. All participants provided written informed consent and were free to withdraw without penalty. No personally identifiable data were collected; all results are reported at aggregate level only.

## Results

**Figure 1:**
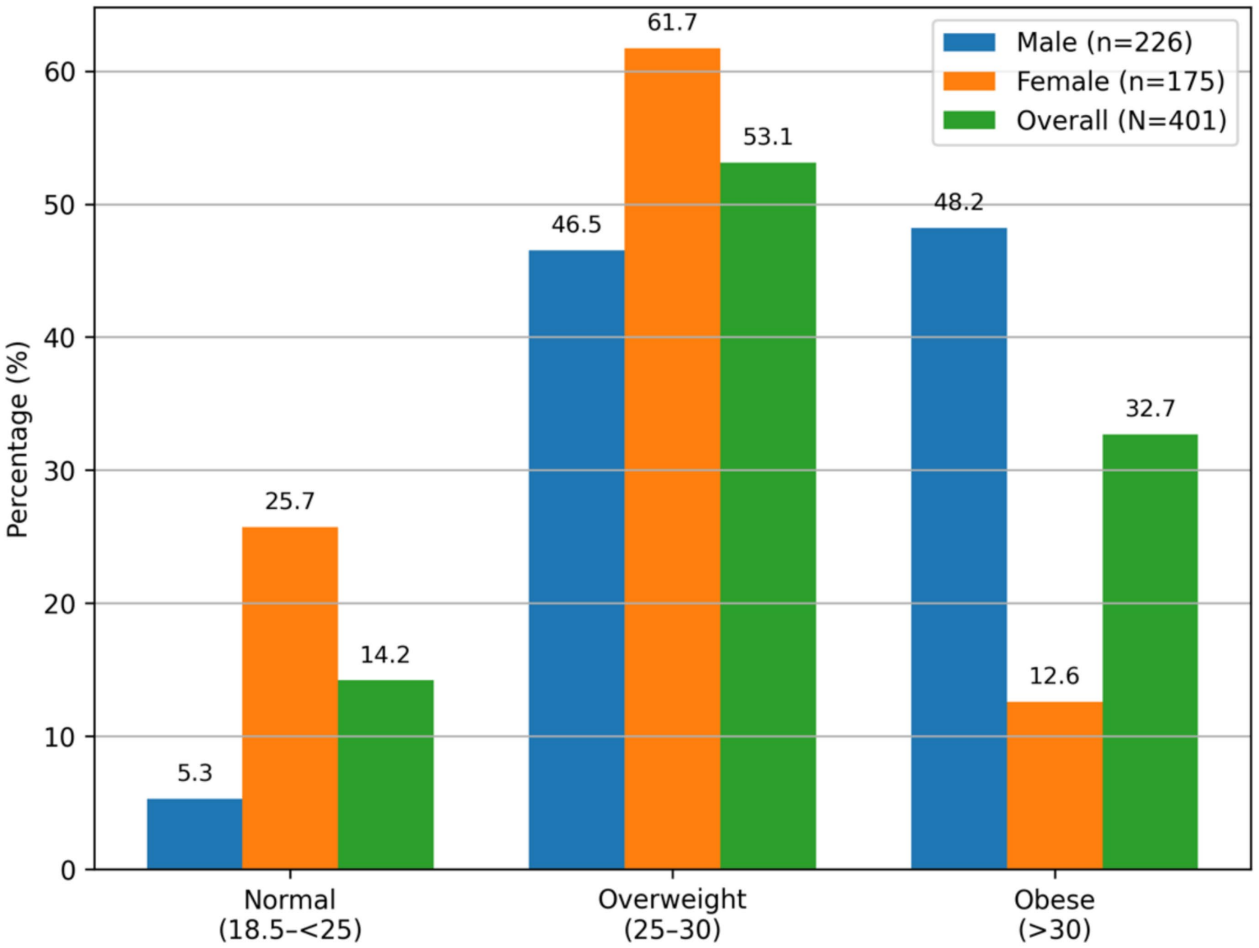
BMI distribution (%) by sex among academic staff at the University of Calabar, Nigeria (*N* = 401). Values represent column percentages within each sex group. BMI, body mass index.

Of 401 participants, 56.4% were male (*n* = 226) and 43.6% female (*n* = 175). The mean age was 41.53 years (SD = 8.50; range 27–70). Most held a PhD or equivalent qualification (51.9%) and were married (69.8%). By academic rank, Lecturer I was the most common category (22.4%), whilst Professors constituted the smallest group (7.0%). Nearly 70% (69.8%) concurrently held administrative positions within UNICAL. Mean systolic and diastolic blood pressures were 121.78 (SD = 11.17) and 77.05 (SD = 7.70) mmHg, respectively; maximum values of 152/100 mmHg indicated that a subset were hypertensive at measurement. Mean number of independently taught courses was 1.34 (SD = 2.03), and mean class size was 282 students (SD = 29; range 10–1,500). Full participant characteristics are shown in Table 1. Table 2 documents lifestyle behaviours stratified by ASSET stress category. The most clinically meaningful patterns appear in alcohol use and physical activity: respondents classified in the high-stress category reported substantially higher rates of frequent alcohol consumption 18.3% drinking three to six times per week and 15.0% drinking daily or more compared with 5.6 and 1.8%, respectively, in the moderate-stress group. High-stress respondents were also far less likely to engage in vigorous exercise (3.3% vs. 18.6%). Both associations reached statistical significance (alcohol frequency: χ^2^ = 9.84, *p* = 0.043; exercise level: χ^2^ = 11.27, *p* = 0.024), lending empirical support to the stress-health behaviour pathway through which chronic occupational stress disrupts health-protective behaviours and may progressively elevate cardiometabolic risk (4, 5, 26). Table 3 presents self-reported health status across the full sample. The apparent discordance between predominantly positive health self-ratings (97.5% reporting good health or better) and the simultaneously high prevalence of overweight, obesity, and hypertension medication use is worth unpacking. Self-rated health reflects holistic functional appraisal how well someone feels they are coping and performing rather than direct clinical measurement. Most respondents remained professionally active and physically engaged, which plausibly sustains positive self-appraisal even in the presence of subclinical morbidity (14). Hypertension was the dominant chronic condition, accounting for 48.4% of all medication mentions among the 18.5% on long-term treatment, a prevalence consistent with the established links between sustained occupational stress and cardiovascular risk in resource-constrained settings. Table 4 profiles perceived burden across the 12 ASSET organisational domains adapted from Cartwright and Cooper (10). Pay and benefits attracted the sharpest extreme response, with 19.5% of respondents rating it a very strong burden the highest such frequency across the entire instrument. Work overload generated the broadest combined strong-to-very-strong burden rating at 36.4%, and work-life balance was most commonly rated a moderate burden (46.9%). Taken together, the distribution points to a workforce under sustained pressure concentrated in structural and resource-related domains, a pattern that maps directly onto the Job Demands-Resources model’s prediction that burnout and strain follow when job demands chronically outpace available resources (2). Table 5 captures respondents’ priority rankings of stressors affecting job productivity. Work overload dominated first-ranked nominations at 15.2%, almost three times the frequency of the next stressor (pay and benefits, 5.5%), and retained its primacy at second rank (10.2%). The sharp drop at third rank (1.0%) confirms that respondents who identified workload as a stressor regarded it as their primary not incidental concern. Resources and communication was the second most frequently cited second-ranked stressor (9.5%), suggesting that infrastructure deficits compound and amplify the overload problem. These patterns align closely with findings from comparable Nigerian academic populations (7, 8) and with international faculty stress evidence (15). Table 6 applies the same ranking structure to job satisfaction stressors and reveals a notably different profile. Inadequate pay and benefits dominated first-ranked citations at 17.7% a frequency more than four times any other first-rank nominee whilst work overload barely registered at first rank (0.2%). At third rank, workplace conditions emerged as the leading concern (7.0%), followed by resources and communication (4.2%). The divergence between Tables 5, 6 is analytically meaningful: workload primarily impairs functional performance, whilst financial inadequacy and poor infrastructure erode professional motivation and the affective dimensions of job commitment two mechanistically distinct pathways that require correspondingly distinct institutional responses (2, 16, 27). Table 7 summarises the full ASSET score distribution using the empirically validated thresholds of Faragher et al. (11) and Cartwright and Cooper (10). The overall mean of 192.04 (SD = 26.49) places the sample within the moderate stress band, but the distribution is heavily skewed towards its upper boundary: 84.3% scored in the moderate range and 15.0% in the high range, leaving only three respondents (0.8%) in the low category. In practical terms, low workplace stress is essentially absent from this institution. That figure sits notably above what comparative studies have reported for academic staff in higher-income countries (17, 18), reinforcing the argument that structural conditions in Nigerian public universities generate stress levels qualitatively different in scale from those observed in better-resourced settings. Table 8 tests associations between four socio-demographic variables and self-reported overall workplace stress rated on a five-point scale. All four variables reached significance at *p* < 0.001. Female academics more frequently endorsed strong stress (36.0% vs. 21.2% in males), whilst males more commonly reported very strong stress (15.9% vs. 1.1%), a divergence that likely reflects both genuine gender differences in stress expression and the dual burden of professional and domestic responsibilities disproportionately carried by women in this context (19). PhD holders showed the highest combined strong-to-very-strong stress rate (47.6%). The non-linear pattern by teaching experience is arguably the table’s most striking feature: stress peaks sharply at 7–9 years and again at 13–15 years, identifying specific mid-career windows not early or late career as the periods of greatest vulnerability (15, 20). Table 9 repeats this analysis substituting ASSET-derived categories for subjective stress ratings and reveals one important revision. The gender association, significant in Table 8, does not survive the switch to psychometric measurement (χ^2^ = 3.86, *p* = 0.145), a discrepancy that underscores a genuine conceptual distinction: self-appraised stress and standardised instrument-derived stress scores are not interchangeable, and treating them as such risks methodological confusion. Educational level, academic rank, and teaching experience all retained highly significant associations (all p < 0.001). The 7-9-year experience group produced the most striking result: 40.2% fell in the high-stress category, more than double the proportion in any adjacent experience group a finding that places mid-career academics at the institutional epicentre of the stress problem (15, 21). Table 10 tests the cross-sectional association between BMI category and ASSET stress level and returns a non-significant result (χ^2^ = 2.17, df = 4, *p* = 0.704). Across all three BMI groups, the distribution of stress categories was broadly similar, with moderate stress predominating. That said, interpreting this null finding as evidence that stress and body weight are unrelated would be a mistake. The stress-obesity relationship operates primarily through cumulative, longitudinal mechanisms HPA axis dysregulation, chronic cortisol elevation, sleep disruption, and the progressive degradation of health-promoting behaviours documented in Table 2 none of which a single cross-sectional measurement is designed to capture (4, 5). The absence of a significant association at one point in time is entirely compatible with a meaningful relationship unfolding over years. Analysis of BMI categories revealed sex differences in weight status (Figure 1). Overall, 53.1% of respondents were classified as overweight. Among male staff (*n* = 226), 48.2% were obese and 46.5% were overweight, with only 5.3% within the normal BMI range. Female staff (*n* = 175) showed a more favourable distribution, with the majority classified as overweight (61.7%), a substantially lower proportion obese (12.6%), and a higher proportion within the normal BMI range (25.7%) compared with males. Obesity was therefore disproportionately prevalent among male academic staff.

**Table 1:** Socio-demographic, anthropometric, physiological, and professional characteristics (*N* = 401).

Variable	Category	*n* (%)
Gender	Male	226 (56.4)
	Female	175 (43.6)
Educational level	Bachelor’s degree	29 (7.2)
	Master’s degree	164 (40.9)
	PhD or equivalent	208 (51.9)
Academic rank	Professor	28 (7.0)
	Associate professor	51 (12.7)
	Senior lecturer	85 (21.2)
	Lecturer I	90 (22.4)
	Lecturer II	72 (18.0)
	Assistant lecturer	40 (10.0)
	Graduate assistant	35 (8.7)
Total teaching experience	1–3 years	76 (19.0)
	4–6 years	105 (26.2)
	7–9 years	112 (27.9)
	10–12 years	54 (13.5)
	13–15 years	35 (8.7)
	≥16 years	19 (4.7)

Anthropometric/physiological (means)	M (SD)	Range
Age (years)	41.53 (8.50)	27–70
Systolic BP (mmHg)	121.78 (11.17)	100–152
Diastolic BP (mmHg)	77.05 (7.70)	52–100
Waist circumference (cm)	95.78 (11.12)	30–115
Hip circumference (cm)	102.34 (9.94)	36–122

BP = blood pressure; M = mean; SD = standard deviation. BMI distribution is presented in Figure 1.

**Table 2:** Lifestyle characteristics by ASSET stress category (*N* = 401).

Variable	Total *n* (%)	Low *n* (%)	Moderate *n* (%)	High *n* (%)
Smoking				
Yes	11 (2.7)	0 (0.0)	9 (2.7)	2 (3.3)
No	390 (97.3)	3 (100)	329 (97.3)	58 (96.7)
Alcohol consumption				
Yes	243 (60.6)	2 (66.7)	197 (58.3)	44 (73.3)
No	158 (39.4)	1 (33.3)	141 (41.7)	16 (26.7)
Alcohol frequency*				
1–2 times/week	198 (49.4)	2 (66.7)	172 (50.9)	24 (40.0)
3–6 times/week	30 (7.5)	0 (0.0)	19 (5.6)	11 (18.3)
≥7 times/week	15 (3.7)	0 (0.0)	6 (1.8)	9 (15.0)
Physical exercise				
Yes	357 (89.0)	3 (100)	303 (89.6)	51 (85.0)
No	44 (11.0)	0 (0.0)	35 (10.4)	9 (15.0)
Exercise level†				
High	67 (16.7)	2 (66.7)	63 (18.6)	2 (3.3)
Moderate	182 (45.4)	1 (33.3)	161 (47.6)	20 (33.3)
Low	105 (26.2)	0 (0.0)	76 (22.5)	29 (48.3)

ASSET = A Shortened Stress Evaluation Tool; categories: low (≤142), moderate (143–231), high (≥232). *Restricted to current drinkers. †Restricted to physically active respondents. Chi-square association with ASSET category: alcohol frequency χ^2^ = 9.84, *p* = 0.043; exercise level χ^2^ = 11.27, *p* = 0.024.

**Table 3:** Self-reported health status of respondents (*N* = 401).

Variable	*N*	%
Self-rated health		
Excellent	42	10.5
Very good	231	57.6
Good	118	29.4
Fair	6	1.5
Poor	4	1.0
Regular health monitoring		
Yes	299	74.6
No	102	25.4
Health problem in preceding 3 months		
Yes	92	22.9
No	309	77.1
Currently on chronic medication		
Yes	74	18.5
No	327	81.5
Condition for which medication was taken (*n* = 95 mentions)^ᵃ^		%^ᵇ^
Hypertension	46	48.4
Anaemia	15	15.8
Diabetes mellitus	6	6.3
Liver disease	6	6.3
Stroke	4	4.2
Other conditions	18	18.9

ᵃ Multiple-response item; 74 respondents reported 95 condition mentions in total.

ᵇ Percentages calculated from total condition mentions (*n* = 95).

**Table 4:** Perceived occupational burden across 12 stress domains (*N* = 401).

Stress domain	Not at all *n* (%)	Fairly *n* (%)	Moderately *n* (%)	Strongly *n* (%)	Very strongly *n* (%)
Work relationships	69 (17.2)	164 (40.9)	111 (27.7)	31 (7.7)	26 (6.5)
Non-work relationships	130 (32.4)	82 (20.4)	136 (33.9)	45 (11.2)	8 (2.0)
Work–life balance	50 (12.5)	68 (17.0)	188 (46.9)	87 (21.7)	8 (2.0)
Work overload	59 (14.7)	38 (9.5)	158 (39.4)	92 (22.9)	54 (13.5)
Job security	95 (23.7)	108 (26.9)	86 (21.4)	94 (23.4)	18 (4.5)
Control over work	69 (17.2)	142 (35.4)	114 (28.4)	61 (15.2)	15 (3.7)
Resources and communication	80 (20.0)	89 (22.2)	106 (26.4)	100 (24.9)	26 (6.5)
Pay and benefits	67 (16.7)	41 (10.2)	158 (39.4)	57 (14.2)	78 (19.5)
Workplace conditions	68 (17.0)	83 (20.7)	94 (23.4)	109 (27.2)	47 (11.7)
Workplace safety/security	69 (17.2)	111 (27.7)	139 (34.7)	65 (16.2)	17 (4.2)
Institutional values	89 (22.2)	54 (13.5)	166 (41.4)	73 (18.2)	19 (4.7)
Organisational commitment	70 (17.5)	94 (23.4)	133 (33.2)	84 (20.9)	20 (5.0)

Adapted from Cartwright and Cooper (10).

**Table 5:** Priority rankings of stressors affecting job productivity (*N* = 401).

Stressor	*N*	%
First-ranked		
Work overload	61	15.2
Pay and benefits	22	5.5
Workplace conditions	17	4.2
Work relationships	15	3.7
Organisational commitment	15	3.7
Job security	11	2.7
Second-ranked		
Work overload	41	10.2
Resources and communication	38	9.5
Work–life balance	17	4.2
Job security	15	3.7
Work relationships	3	0.7
Third-ranked		
Workplace conditions	26	6.5
Pay and benefits	21	5.2
Organisational commitment	21	5.2
Work–life balance	20	5.0
Non-work relationships	15	3.7

Percentages reflect proportions of total sample (*N* = 401) selecting each stressor at the stated rank position.

**Table 6:** Priority rankings of stressors affecting job satisfaction (*N* = 401).

Stressor	*n*	%
First-ranked		
Pay and benefits	71	17.7
Non-work relationships	16	4.0
Job security	15	3.7
Workplace conditions	5	1.2
Work relationships	4	1.0
Second-ranked		
Work overload	18	4.5
Work relationships	15	3.7
Resources and communication	15	3.7
Pay and benefits	15	3.7
Institutional values	14	3.5
Third-ranked		
Workplace conditions	28	7.0
Resources and communication	17	4.2
Work overload	16	4.0
Organisational commitment	16	4.0
Work–life balance	15	3.7

Percentages reflect proportions of total sample (*N* = 401).

**Table 7:** Distribution of ASSET total score categories (*N* = 401).

Stress category	Score range	*n*	%
Low	≤142	3	0.8
Moderate	143–231	338	84.3
High	≥232	60	15.0

ASSET = A Shortened Stress Evaluation Tool (10, 11). Categorisation thresholds derived from a normative database of >9,000 employees. Overall ASSET score: M = 192.04, SD = 26.49; range: 112–241.

**Table 8:** Association between socio-demographic variables and self-reported overall stress level.

Variable	Not at all *n* (%)	Fairly *n* (%)	Moderately *n* (%)	Strongly *n* (%)	Very strongly *n* (%)	df	χ^2^ (*p*)
Gender							
Male	16 (7.1)	29 (12.8)	97 (42.9)	48 (21.2)	36 (15.9)	4	35.60 (<0.001)
Female	15 (8.6)	11 (6.3)	84 (48.0)	63 (36.0)	2 (1.1)		
Educational level							
Bachelor’s	1 (3.4)	7 (24.1)	10 (34.5)	10 (34.5)	1 (3.4)	8	58.85 (<0.001)
Master’s	16 (9.8)	29 (17.6)	77 (46.9)	40 (24.4)	2 (1.2)		
PhD/equiv.	15 (7.2)	4 (1.9)	90 (43.3)	63 (30.3)	36 (17.3)		
Teaching experience							
1–3 years	1 (1.3)	9 (11.8)	44 (57.9)	20 (26.3)	2 (2.6)	20	92.96 (<0.001)
4–6 years	2 (1.9)	13 (12.4)	60 (57.1)	14 (13.3)	16 (15.2)		
7–9 years	15 (13.4)	14 (12.5)	27 (24.1)	41 (36.6)	15 (13.4)		
10–12 years	12 (22.2)	3 (5.5)	21 (38.9)	17 (31.5)	1 (1.9)		
13–15 years	1 (2.9)	1 (2.8)	14 (40.0)	18 (51.4)	1 (2.9)		
≥16 years	1 (5.3)	4 (21.0)	8 (42.1)	1 (5.3)	5 (26.3)		

χ^2^ statistics are shown for the first data row of each variable group. Overall stress rating was assessed on a five-point scale from “not at all” to “very strongly”.

**Table 9:** Association between socio-demographic characteristics and ASSET stress category.

Variable	Low *n* (%)	Moderate *n* (%)	High *n* (%)	df	χ^2^ (*p*)
Gender					
Male	0 (0.0)	196 (86.7)	30 (13.3)	2	3.86 (0.145)
Female	3 (1.7)	142 (81.1)	30 (17.1)		
Educational level					
Bachelor’s	3 (10.3)	24 (82.8)	2 (7.4)	4	41.85 (<0.001)
Master’s	0 (0.0)	149 (90.9)	15 (9.1)		
PhD/equiv.	0 (0.0)	163 (78.4)	45 (21.6)		
Academic rank					
Professor	0 (0.0)	26 (92.9)	2 (7.1)	12	47.05 (<0.001)
Assoc. professor	0 (0.0)	39 (76.5)	12 (23.5)		
Senior lecturer	0 (0.0)	65 (76.5)	20 (23.5)		
Lecturer I	0 (0.0)	74 (82.2)	16 (17.8)		
Lecturer II	0 (0.0)	60 (83.3)	12 (16.7)		
Asst. lecturer	0 (0.0)	38 (95.0)	2 (5.0)		
Grad. assistant	3 (8.5)	29 (82.9)	3 (8.6)		
Teaching experience					
1–3 years	3 (4.0)	71 (93.4)	2 (2.6)	10	112.28 (<0.001)
4–6 years	0 (0.0)	100 (95.2)	5 (4.7)		
7–9 years	0 (0.0)	67 (59.8)	45 (40.2)		
10–12 years	0 (0.0)	39 (72.2)	15 (27.8)		
13–15 years	0 (0.0)	32 (91.4)	3 (8.5)		
≥16 years	0 (0.0)	17 (89.4)	2 (10.5)		

ASSET categories: low (≤142), moderate (143–231), high (≥232).

**Table 10:** Association between BMI category and ASSET stress level (*N* = 401).

BMI category	Low *n*	Moderate *n*	High *n*	df	χ^2^ (*p*)
Normal (18.5–<25 kg/m^2^)	1	50	6	4	2.17 (0.704)
Overweight (25–≤30 kg/m^2^)	1	180	32		
Obese (>30 kg/m^2^)	1	108	22		

BMI = body mass index. ASSET stress levels: low (≤142), moderate (143–231), high (≥232).

## Discussion

This study examined the prevalence, organisational sources, and health and occupational consequences of workplace stress among academic staff at UNICAL. The scale of the problem is, quite frankly, stark. With 99.3% of respondents recording moderate-to-high ASSET scores, near-universal workplace stress appears to be the structural condition of academic work in this setting not an outlier or individual failing, but an institutional norm. This is consistent with the pattern reported across sub-Saharan African universities (7, 8) and mirrors broader international evidence on academic occupational stress (15, 17, 18).

The socio-demographic patterning of stress revealed some interesting, and arguably counterintuitive, findings. Female academics reported higher rates of strong self-reported stress than male counterparts, which I’d argue reflects the well-documented dual burden of professional and domestic responsibilities that disproportionately falls on women in academic settings a pattern observed in Nigerian and wider West African contexts (19). Yet this gender difference did not reach significance using the ASSET-derived category (*p* = 0.145), pointing to an important methodological distinction: subjective stress appraisal and psychometrically measured stress scores are not interchangeable constructs.

Mid-career academics specifically Associate Professors and Senior Lecturers with doctoral qualifications and seven to twelve years of experience showed the highest ASSET scores. This follows a recognisable pattern in the international faculty stress literature. In a way, the mid-career period is a structural pinch point: the expectations of a full professor apply without the security of tenure or the protected status of early-career mentorship. Publication targets, grant competition, teaching loads, and administrative responsibilities converge precisely when personal commitments family, children, mortgages tend to peak. Padilla and Thompson (20) described this dynamic, and Sabagh et al. (15) reinforced it empirically. That early-career staff in this sample showed comparatively lower stress is, arguably, less reassuring than it might appear: it may simply reflect sheltered expectations rather than a genuinely manageable workload.

The stressor landscape was dominated by work overload, inadequate pay and benefits, poor resources and communication, and adverse workplace conditions—precisely the structural features that define under-resourced public universities in low- and middle-income countries. These findings align closely with the Job Demands-Resources framework (2), which predicts that burnout and performance deterioration occur when job demands consistently outpace available resources. In Nigerian universities, that gap is wide and, based on available evidence, widening (7). Respondents who reported stronger organisational commitment and alignment with institutional values were less likely to fall in the high stress category, consistent with Social Exchange Theory (16): where perceived organisational investment is high, employees tend to reciprocate with resilience and engagement.

Perhaps the most analytically significant contribution of this study is the explicit linking of lifestyle behaviours to stress levels something prior Nigerian studies have largely not done. Respondents in the high ASSET category were significantly more likely to drink alcohol frequently (χ^2^ = 9.84, *p* = 0.043) and to report low or no physical activity (χ^2^ = 11.27, *p* = 0.024). This is not surprising from a mechanistic standpoint: chronic stress dysregulates the hypothalamic–pituitary–adrenal (HPA) axis, elevates cortisol, disrupts sleep, and depletes the motivational resources needed to maintain health-promoting behaviours (4, 5). The result, over time, is a plausible pathway to elevated BMI, visceral adiposity, and cardiometabolic risk. The absence of a significant cross-sectional association between BMI category and ASSET stress level (χ^2^ = 2.17, *p* = 0.704) does not contradict this pathway—it simply reflects the time horizon mismatch inherent in cross-sectional designs. The stress-obesity relationship is almost certainly bidirectional and accumulates over years, not months (4).

On health outcomes, the finding that respondents in the high ASSET category showed higher rates of hypertension medication use (23.3% vs. 16.9% in the moderate category) is clinically important and consistent with the established stress-cardiovascular disease literature (4, 5). That 18.5% of the sample was on chronic medication, with hypertension accounting for nearly half of all condition mentions, warrants institutional attention. In Nigeria, hypertension is frequently underdiagnosed and inadequately treated, with compounding effects on work capacity and attendance (22). The coexistence of generally positive self-rated health with objectively elevated BMI and hypertension prevalence reflects a well-documented phenomenon: self-rated health captures holistic functional appraisal, social role performance, and comparative self-positioning within peer groups, not direct clinical parameters (14). Most respondents were professionally active, exercising, and not functionally disabled which, combined with cultural norms around health self-perception in this population, would sustain positive self-ratings even in the presence of subclinical morbidity.

The policy implications extend beyond workload and pay. This study’s findings point to three additional domains that Nigerian universities have largely not engaged with formally: mental health infrastructure, occupational wellness frameworks, and career development architecture. Confidential counselling services, employee assistance programmes, and peer support networks for mid-career academics represent evidence-based interventions with demonstrable efficacy in comparable settings internationally (19, 23). Transparent promotion criteria and equitable workload distribution would target the structural stressors most clearly associated with the 7-12-year experience group. Regular occupational health audits using validated instruments as demonstrated here would enable universities to monitor trends rather than respond only to crises.

### Strengths and limitations

The study’s principal strengths include the use of a psychometrically validated instrument (ASSET) with normatively established thresholds, integration of objective clinical measurements alongside self-report data, the explicit analysis of lifestyle behaviours in relation to stress levels rather than in descriptive isolation, and a diverse sample spanning all 15 faculties, multiple academic ranks, and a broad range of career stages. Limitations are acknowledged. The cross-sectional design means causal inference is not possible. Self-reported outcomes are susceptible to recall bias and social desirability effects. Single-institution sampling limits generalisability to other Nigerian and sub-Saharan African university settings. The analysis of lifestyle-stress associations used cross-sectional chi-square tests; the modest cell sizes in the low-stress group (*n* = 3) constrain the robustness of three-way comparisons. Future longitudinal studies should examine causal pathways between sustained occupational stress and downstream health outcomes. Multi-site comparative studies across Nigerian public universities would strengthen external validity and enable identification of institution-level protective factors.

## Conclusion

Workplace stress is pervasive among academic staff at UNICAL and is not uniformly distributed: mid-career, doctoral-qualified academics bear the heaviest burden. Stress is associated with more frequent alcohol consumption, lower physical activity, and higher rates of hypertension medication use. The primary stressors are structural and institutional—work overload, inadequate remuneration, resource scarcity, and poor workplace conditions—and require systemic responses rather than individual-level coping strategies. Universities should conduct periodic occupational stress audits using validated instruments; establish accessible, confidential mental health and counselling services; develop evidence-based occupational wellness frameworks; and create transparent career development pathways that acknowledge the particular pressures of the mid-career stage. Equitable remuneration and sustainable workload management remain foundational. Contextually adapted stress management interventions for the sub-Saharan African academic environment represent a pressing research and policy priority (24).

## Data Availability

The original contributions presented in the study are included in the article/supplementary material, further inquiries can be directed to the corresponding author.
